# Spatial-Temporal Characteristics of Brain Activity in Autism Spectrum Disorder Based on Hidden Markov Model and Dynamic Graph Theory: A Resting-State fMRI Study

**DOI:** 10.3390/brainsci14050507

**Published:** 2024-05-17

**Authors:** Shiting Qian, Qinqin Yang, Congbo Cai, Jiyang Dong, Shuhui Cai

**Affiliations:** Fujian Provincial Key Laboratory of Plasma and Magnetic Resonance, Department of Electronic Science, Xiamen University, Xiamen 361005, China; 33320211150265@stu.xmu.edu.cn (S.Q.); qqyang@stu.xmu.edu.cn (Q.Y.); cbcai@xmu.edu.cn (C.C.); jydong@xmu.edu.cn (J.D.)

**Keywords:** functional magnetic resonance imaging, autism spectrum disorder, brain network, functional connectivity, hidden Markov model, dynamic graph theory

## Abstract

Autism spectrum disorder (ASD) is a common neurodevelopmental disorder. Functional magnetic resonance imaging (fMRI) can be used to measure the temporal correlation of blood-oxygen-level-dependent (BOLD) signals in the brain to assess the brain’s intrinsic connectivity and capture dynamic changes in the brain. In this study, the hidden Markov model (HMM) and dynamic graph (DG) theory are used to study the spatial-temporal characteristics and dynamics of brain networks based on dynamic functional connectivity (DFC). By using HMM, we identified three typical brain states for ASD and healthy control (HC). Furthermore, we explored the correlation between HMM time-varying properties and clinical autism scale scores. Differences in brain topological characteristics and dynamics between ASD and HC were compared by DG analysis. The experimental results indicate that ASD is more inclined to enter a strongly connected HMM brain state, leading to the isolation of brain networks and alterations in the topological characteristics of brain networks, such as default mode network (DMN), ventral attention network (VAN), and visual network (VN). This work suggests that using different data-driven methods based on DFC to study brain network dynamics would have better information complementarity, which can provide a new direction for the extraction of neuro-biomarkers in the early diagnosis of ASD.

## 1. Introduction

Autism spectrum disorder (ASD) is a common heterogeneous neurodevelopmental disorder affecting adolescents, with symptoms characterized by a persistent lack of social communication, repetitive and restricted patterns of behaviors, interests, or activities [1,2], and susceptibility to other comorbid disorders, such as depression, mental retardation, and deficient pre-attentive discrimination [3,4], which can jeopardize physical and psychological health. Current clinical treatments are limited, especially in early clinical therapeutic interventions [5,6]. Functional magnetic resonance imaging (fMRI) can be used to detect intrinsic brain connectivity by calculating the temporal correlation of spontaneous blood-oxygen-level-dependent (BOLD) signals between spatially distributed regions of interest (ROI) in the brain [7]. By understanding brain network activity, we can assess changes in brain dynamics in ASD, which would help study this population [8].

Cognitive function has very complex relationships with specific regions in the brain. Previous studies have constructed functional brain networks using functional connectivity (FC) and resting state fMRI [9]. In 2011, Yeo et al. used a cluster analysis algorithm to classify seven resting-state brain networks in the cerebral cortex [10]. Among them, the most closely related to ASD symptoms include (1) the visual network (VN), which processes visual information; (2) the ventral attention network (VAN), which is an essential mediator of stimulus-driven attention [11]; (3) the default mode network (DMN), which is a large-scale network with high functional connectivity that includes a set of brain regions contributing to social and self-referential cognitive processes [12]. The relationship between cognitive and behavioral functions and brain regions is a hot topic in the research of ASD.

The alteration of FC in the brain is one of the hallmarks of ASD [13]. In recent years, researchers have studied brain FC in ASD, providing valuable insights into brain dysfunction and clinical significance. Tong et al. combined contrastive learning and sparse canonical correlation analysis to identify the dimensions of resting-state electroencephalographic (EEG) connectivity in ASD, which were significantly correlated with social and communication deficits (SCDs) and restricted and repetitive behaviors (RRBs) [14]. Jain et al. explored the impact of brain FC patterns on the diagnosis of ASD using deep neural networks (DNNs) and developed proper diagnostic models to address ASD heterogeneity [15]. Although FC is based on the assumption that neural signal fluctuations have a time-invariant nature, the spontaneous fluctuations in brain activity and the dynamics of brain networks should be noted [16,17].

The smooth, continuous transitions between brain states are directly related to cognitive function. Since the fMRI is inherently dynamic, more and more studies have begun to analyze dynamic functional connectivity (DFC) to explore ASD [18]. DFC refers to the FC that changes quickly, which can effectively give feedback on the time-varying characteristics of FC over time [19]. He et al. found that the social cognitive dysfunction of ASD was related to the dysfunction of the DMN by studying the DFC abnormalities between children with ASD and normal controls [20]. Guo et al. found that the temporal dynamics of intra-hemispheric and interhemispheric functional connectivity were altered in brain regions with ASD and had a potential impact on impaired social functioning in ASD [21]. The DFC has strong robustness in identifying subtle disease-induced neuro-functional wiring disruptions and can potentially be an effective biomarker for brain disease diagnosis [22,23]. The most common DFC analysis technique is the sliding window-based method, in which the accuracy of the analysis is significantly affected by the step size and sliding window width [24].

The research on the spatial-temporal properties of brain networks have been increasingly reported. Jia et al. analyzed the abnormal spatial-temporal characteristics of cerebral cortical activity in patients with ASD by conducting microstate analysis of resting state EEG [25]. Smith et al. found the imbalance of spatial-level connectivity and dynamic temporal entropy in ASD brain networks based on machine learning methods [26]. Qiao et al. achieved early prediction of ASD based on multivariate spatial-temporal brain characteristics [27,28]. The in-depth study of the spatial-temporal characteristics of the brain networks is significant for the future diagnosis and pathological interpretation of ASD.

In recent years, the hidden Markov model (HMM) based on probabilistic generative models describes brain activity as a dynamic sequence of brain states discretized on the time scale of data evaluation. The basic assumption of HMM is that for each time point, the state variable determines the probability that each state is activated at that instant, so we can determine which state is activated based on the maximum likelihood of each state [29,30]. Namely, HMM describes a hidden process that depends only on the current state and not any previous state, which can obtain the probability of occurrence of each state and the corresponding brain region activity intensity at a specific time section. The model selection (number of hidden states) and the learning of HMM parameters are data-driven. It is an effective method to study the DFC based on the HMM state. Applying HMM to fMRI-based brain network construction can effectively overcome the limitations of sliding window methods and provide a more plentiful temporal description in a smaller time scale [31,32]. Jun et al. distinguished ASD and typical development subjects based on HMM and explored the functional network characteristics of BOLD time signals based on HMM’s hidden state sequences [33]. Lin et al. used HMM to reconfigure the ASD brain network and characterize the temporal specificity of brain activity on fMRI data [34]. However, in existing research, the short-distance dependence of HMM states, together with the number of HMM states and parameters, constitutes a limit to the accurate interpretation of brain states.

Additionally, graph theory analysis based on nodes and edges has been increasingly applied to fMRI [35]. Graph theory is used to model pairwise relationships between variables and explore the associations between interacting ROIs in complex networks, which is suitable for comprehensive studies of whole-brain network characteristics [36]. Dynamic graph (DG) theory based on DFC can reveal topological features in brain space information and provide potential neuro-biomarkers as targets for the early diagnosis and recognition of diseases [37]. The global and nodal attributes define connectional attributes of brain regions and derive spatial-temporal variability of brain network topological properties [38,39]. Jafadideh et al. studied the influence of DFC on the DG global indexes of ASD and HC. They concluded that topological analysis of DFC states could provide discriminative features unavailable in static FC and structural connectivity [40]. Jiao et al. constructed dynamic brain networks using the sliding time window method and analyzed the graph theory indicators reflecting the FC state to compare the brain network differences between mild cognitive impairment (MCI) and normal controls [41]. DG metrics and the characteristics of the brain network can also be used to classify ASD and HC [42]. However, as the temporal instability of topological features, the functional connectivity is non-stationary, resulting in different topological structures of brain networks obtained by various methods.

Therefore, it is worth considering to adopt a joint application of HMM and DG theory in the construction of a dynamic brain network and the comprehensive description of spatial-temporal characteristics in ASD brains to seek a thorough explanation of brain activities. The exploration based on different dimensions is expected to achieve complementary interpretation of brain information, comprehensively analyze the heterogeneity of the ASD brain network, and overcome the respective limitations of the two methods.

In this work, we applied data-driven HMM and DG theory methods on the ABIDE (Autism Brain Imaging Data Exchange) dataset to characterize the brain networks’ dynamic changes and functional connectivity. The spatial-temporal variability of the topological structure is extracted by DG theory analysis. The abnormal connectivity network associated with ASD is established. The HMM provided a detailed description of the temporal characteristics of brain dynamics and offset the shortcomings of the DG theory method. We investigated the correlation between HMM indicators and clinical autism scale scores to discern pattern differences. The dynamic changes and spatial-temporal characteristics of brain networks were well explained. The analysis at the whole brain level with different approaches improves the accuracy and time-varying robustness of ASD diagnosis and the complementarity of information, providing new insights into the neural mechanism of ASD and the research or application of fMRI in neuroscience.

## 2. Materials and Methods

### 2.1. Participants

The datasets from ABIDE I (http://fcon_1000.projects.nitrc.org/indi/abide/, accessed on 10 June 2023) were used for analysis. ABIDE is an open-access multi-site image repository, and the datasets were collected from different sites using a variety of scanner protocols, including structural and functional scans of ASDs and matched typical development controls. In this study, the subjects were selected based on the following criteria: (1) complete availability of functional and structural image data; (2) complete information on the autism diagnostic interview-revised (ADI-R) and autism diagnostic observation schedule (ADOS) scores, which are the “gold standard” for assessing children with autism [43]; (3) a full intelligence quotient (FIQ, as estimated by the Wechsler simplified scale of intelligence-IV (WASI-IV)) score of 70 or higher; (4) under the age of 18. As a result, the resting-state fMRI data of 184 subjects from five sites (SDSU, KKI, STANFORD, UCLA_1, UCLA_2) were selected, including 89 subjects with ASD and 95 subjects with healthy control (HC). Each site confirmed ASD diagnosis by clinical judgment and standard diagnostic tools (ADOS and ADI-R). Specifically, ADI-R is a behavioral performance score for children with ASD. ADOS total is the total score that evaluates the interactive ability of ASD. ADOS social is used to assess the social ability of ASD. ADOS communication is used to determine the verbal communication ability of ASD. ADOS Gotham is the standardized severity score for ASD. FIQs were collected using a variety of instruments, and measurements were then set to standardized scales between sites. Seven subjects with abnormal head motion were excluded. Finally, we analyzed 177 subjects (87 ASD and 90 HC). The demographic and clinical characteristics of the subjects included in this study are shown in Table 1.

### 2.2. Data Preprocessing

The toolbox used was implemented in the MATLAB platform. The GRETNA tool (http://www.nitrc.org/projects/gretna, accessed on 16 June 2023) was used for data preprocessing [44]. The preprocessing pipeline removed the first five time points and performed the time slicing with a repetition time (TR) of 2 s. The subjects with significant brain tissue loss or head motion exceeding 1.5 mm and 1.5° were excluded. We co-aligned T1-weighted anatomical images and mean functional images. The spatial normalization was performed to obtain normalized images of the same size and orientation. The fMRI images were resampled to a 3 × 3 × 3 mm^3^ voxel size. A Gaussian kernel with a full width at half height of 6 mm was employed for smoothing, and the linear temporal detrend was performed to improve the signal-to-noise ratio (SNR) in the data. The covariates of white matter (WM), cerebrospinal fluid (CSF) signals, and Friston-24 parameters were regressed out to filter out high-frequency noise and irrelevant frequency bands. The obtained functional images were bandpass filtered at 0.01–0.1 Hz.

### 2.3. DFC Network Construction by HMM

We constructed a dynamic whole-brain functional network from BOLD time series signals using the HMM-MAR toolbox (https://github.com/OHBA-analysis/HMM-MAR, accessed on 16 June 2023) [32]. First, we extracted the time series based on the Dosenbach 160-node mapping, which contained 142 brain ROIs and 18 cerebellar ROIs. FCs between the selected ROIs defined the brain-connected edges. Covariance represents the connection between brain regions, and FC strength between brain regions can be calculated by Pearson’s correlation coefficient as follows [45]:

(1) $$R=\frac{COV}{\sqrt{VAR}}$$

*R* denotes FC strength between brain regions, *COV* denotes covariance matrix, and *VAR* indicates variance.

Second, the HMM states were described as a multivariate Gaussian distribution, including the mean activation distribution and the FC matrix. HMM consists of a state sequence and an observation sequence. The state sequence is a sequence of hidden states transformed according to specific rules in the time series, while the observation sequence is the data observed at each time point. Each state of the HMM has different distribution parameters, corresponding to unique patterns of brain activity that recur in other parts of the time series, and the mean activation distribution represents the average activity level of each brain region. We assume that $x_{t}$ represents the multichannel source signal and *S_t_* represents the hidden state variable at time point *t*, the observation model corresponding to MAR (multivariate autoregressive) model characterizes the distribution of state *K* by parameters $\left(\mu_{K},\sum_{K}\right)$ as follows [32,46]:

(2) $$x_{t}|S_{t}=K\;\sim \;\mathrm{Multivariate}\;\mathrm{Gaussian}\;(\mu_{K}{,\sum }_{K})$$

where $\mu_{K}$ is a vector containing the mean BOLD activation, and $\sum_{K}$ is the covariance matrix codifying the variances and covariances between channels when state *K* is active.

The number of hidden states and the form of the observation model needed to be specified. A goodness-of-fit criterion was used to evaluate the degree of fit of the *K*-value in the HMM. Thus, we estimated HMM states with different values of *K* between 2 and 20 to determine the optimal number of states. The number of states with the lowest free energy value corresponding to the *K*-value was the optimal number of states clustered, indicating that the model fits the data best with the fewest parameters. A higher number of states may lead to episodic states, which may occur in only a few subjects [34].

### 2.4. DFC Time Properties by HMM

We computed the HMM time properties by group estimates of the state distribution parameters in time series to reflect HMM state properties and dynamics. The following three dynamic temporal features were identified for the group difference analysis. Average lifetime (ALF) was the ratio of the total time of consecutive visits to the number of visits in a time series. Fractional occupancy (FO) was the ratio of the time spent visiting each state to the total time. The state switching rate (SR) was the transition rate between two states, indicating the stability of each subject’s brain dynamics [47].

### 2.5. DG Theory Computation

Based on the sliding window method of DFC in the DynamicBC 2.1 software (https://www.nitrc.org/projects/dynamicbc, accessed on 16 June 2023) [48], we partitioned different brain regions according to the Dosenbach160 mapping [30]. The strength of the FC between pairwise regions was calculated by Pearson correlation, and the DFC matrices of different time nodes with a dimension of 160 × 160 were extracted for each time window. The Fisher *z*-transformation was performed on the matrices to improve the normality. We used a window width of 50 s and a step size of one repetition time (TR) for the window movement. It was shown that the DFC states can be correctly identified in as little as 30–60 s of data [49].

DG theory analysis was performed using GRETNA. We chose different sparsity thresholds (ranging from 0.05 to 0.3 in a step of 0.01) to compute the binary network. We used the area under curve (AUC) as input and the properties’ variability in different windows as output. The measured node network metrics were nodal clustering coefficient (the inverse of the average shortest path length between the node and all its neighbors) [36], degree centrality (DC, the sum of the FC strength between the nodes) [50], and betweenness centrality (BC, the frequency with which a given node participates in the shortest path between all possible pairs of nodes) [51]. We also computed the global network metrics, *E*_g_ (the parallel information processing capacity of the entire network) and *E*_loc_ (the average information transmission efficiency within a local subnetwork) [52]. Small-world coefficient γ represents the clustering coefficient, λ indicates the average path length, and σ denotes the network small-world attribute ($\sigma =\frac{\gamma }{\lambda }$). γ > 1 and λ ≈ 1, or $\sigma =\frac{\;\gamma \;}{\;\lambda }\;>\;1.1$, is considered to have the small-world property [53]. We used the BrainNet Viewer (www.nitrc.org/projects/bnv/, accessed on 16 June 2023) to visualize the locations of nodes in the brain that differ between ASD and HC [54].

### 2.6. Statistical Analyses

As the multi-site datasets were used in this study, and the FIQ of ASD patients tended to be lower than that of HC (*p* = 0.0086, *t* = −2.656), we regressed out the scan sites and FIQ to exclude their effects for the robustness of the results during statistical analyses. The two-tailed, two-sample *t*-test was used to reveal the source of DFC differences between groups, and the Bonferroni correction (*p* < 0.05) was set to determine the significance level. Correlation analyses were conducted separately for each group to investigate the relationship between HMM dynamic temporal attributes and behavioral scores (FIQ, ADI-R, ADOS). Group differences in graph theory parameters were analyzed using the two-tailed, two-sample *t*-test with a Bonferroni correction (*p* < 0.05) [55].

## 3. Results

### 3.1. HMM Properties

**Figure 1:**
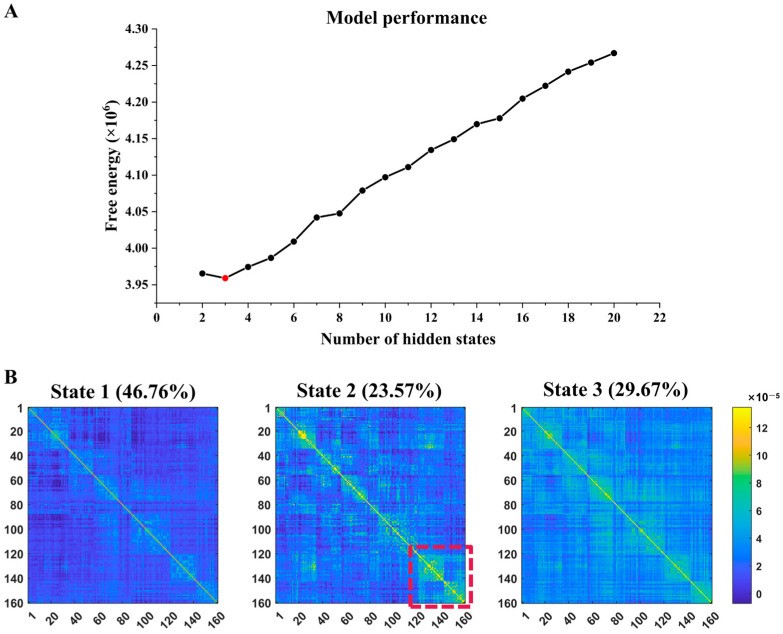
(**A**) Free energy estimation plot (red dot indicates minimum free energy point). (**B**) DFC matrix center-of-mass plots for the three best states are clustered by the HMM. Each state’s average probability of occurrence is given on top of each plot. The horizontal and vertical coordinates represent the ROI numbers based on the Dosenbach160 mapping. The red-dashed box highlights the significant enhancement of DFC in State 2.

**Figure 2:**
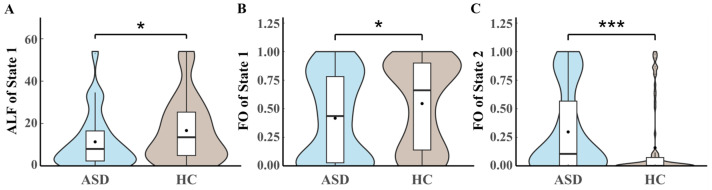
The between-group difference in time-varying characteristics of (**A**) ALF of State 1, (**B**) FO of State 1, and (**C**) FO of State 2. The black dot’s value represents the box plot’s average value. * denotes significance level *p* < 0.05, *** denotes *p* < 0.001. ALF, averaged lifetime; ASD, autism spectrum disorder; FO, fractional occupancy; HC, healthy control.

The free energy results are shown in Figure 1, with the lowest value occurring when the number of states is three. We identified the three best clustering states to yield a relatively high silhouette [56]. State 1 is dominated by weak positive connections, State 2 has progressively stronger connections, and State 3 has the most vital connections, as shown in Figure 2. State 1 has the highest probability of occurrence, followed by State 3. State 2 has the lowest likelihood of occurrence. The connectivity in the lower right corner of the DFC matrix of State 2 is significantly enhanced, indicating that the most significant changes occur in the ventral attention network (VAN), visual network (VN), and subcortical network (SCN) (corresponding to the Yeo 7 network) [57].

### 3.2. HMM Time-Varying Properties

**Figure 3:**
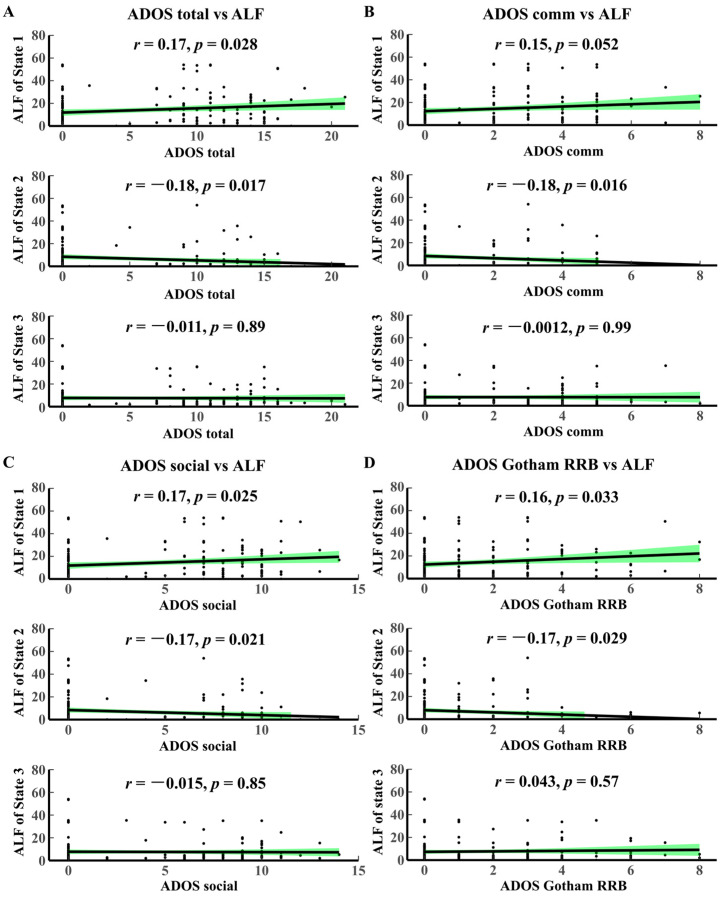
Correlation analysis between ADOS and ALF. (**A**) ADOS total vs. ALF. (**B**) ADOS Gotham vs. ALF. (**C**) ADOS social vs. ALF. (**D**) ADOS Gotham RRB vs. ALF. The black dots represent the value of HMM time-varying characteristic indicators corresponding to different clinical scale scoring values. The green area in each figure represents confidence intervals. ADOS, Autism Diagnostic Observation Schedule; RRB, restricted and repetitive behaviors.

The time-varying characteristics of the ASD brain networks between groups are shown in Figure 3. The ALF of State 1 in ASD is significantly lower than in HC (*p* = 0.0479, *t* = −1.992). The FO of State 1 in ASD is considerably lower than in HC, and the ASD group is not inclined to switch to State 1 (*p* = 0.0236, *t* = −2.283). The FO of State 2 is considerably higher in ASD, meaning that the ASD group is more likely to switch to State 2 (*p* = 0.0067, *t* = 2.731). The results of State 3 and SR do not have significant between-group differences.

### 3.3. Time Properties Correlation

Correlation analyses between the temporal attribute ALF and the ADOS scale (Figure 3) show that (1) ALF State 1 is positively correlated with ADOS total (*p* = 0.028, correlation coefficient r = 0.17), and State 2 is inversely correlated with ADOS total (*p* = 0.017, r = −0.18); (2) ALF State 2 is inversely correlated with ADOS communication (abbreviated as ADOS comm, *p* = 0.016, r = −0.18); (3) ALF State 1 is positively correlated with ADOS social (*p* = 0.028, r = 0.17), and State 2 is inversely correlated with ADOS total (*p* = 0.021, r = −0.17); (4) ALF State 1 is positively correlated with ADOS Gotham RRB (*p* = 0.033, r = 0.16), and ALF State 2 is inversely correlated with ADOS Gotham RRB (*p* = 0.029, r = −0.17).

**Figure 4:**
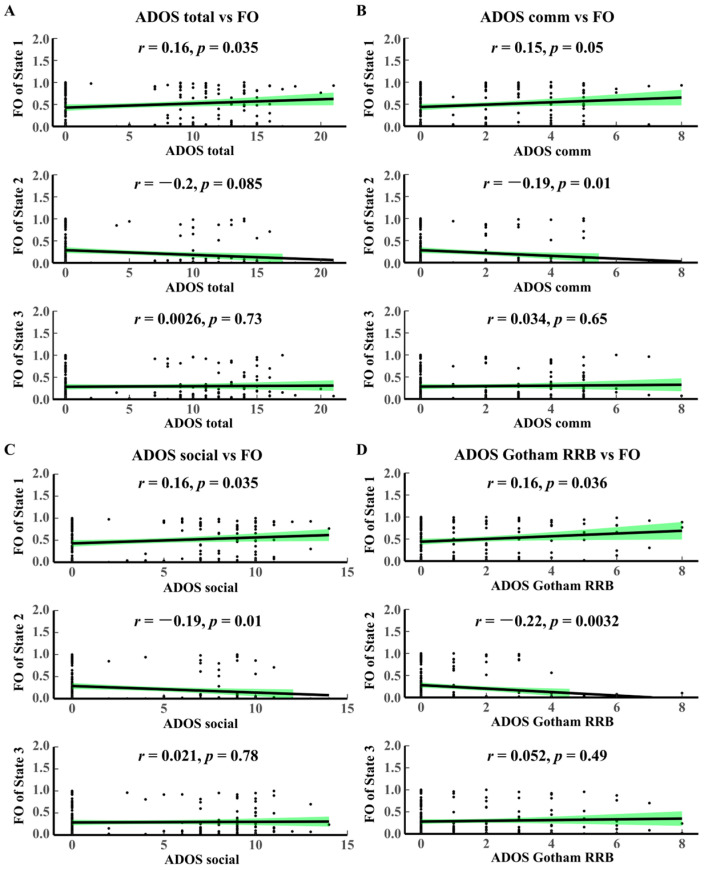
Correlation analysis between ADOS and FO. (**A**) ADOS total vs. FO. (**B**) ADOS comm vs. FO. (**C**) ADOS social vs. FO. (**D**) ADOS Gotham RRB vs. FO. The black dots represent the value of HMM time-varying characteristic indicators corresponding to different clinical scale scoring values. The green area in each figure represents confidence intervals. FO, fractional occupancy.

Correlation analyses of the temporal attribute FO with the ADOS scale (Figure 4) show that (1) FO State 1 is positively correlated with ADOS total (*p* = 0.035, r = 0.16), and FO State 2 is inversely correlated with ADOS total (*p* = 0.0085, r = −0.2); (2) FO State 2 is inversely correlated with ADOS comm (*p* = 0.01, r = −0.19); (3) FO State 1 is positively correlated with ADOS social (*p* = 0.035, r = 0.16), and FO State 2 is inversely correlated with ADOS social (*p* = 0.01, r = −0.19); (4) FO State 1 is positively correlated with ADOS Gotham RRB (*p* = 0.036, r = 0.16), and FO State 2 is inversely correlated with ADOS Gotham RRB (*p* = 0.0032, r = −0.22). The above results reveal a congruent tendency in ALF and FO for the ADOS scale. State 1 is a representative modal feature of HC, and State 2 is a representative modal feature of ASD. The higher the ADOS scale score, the more frequently State 1 occurs in the brain.

**Figure 5:**
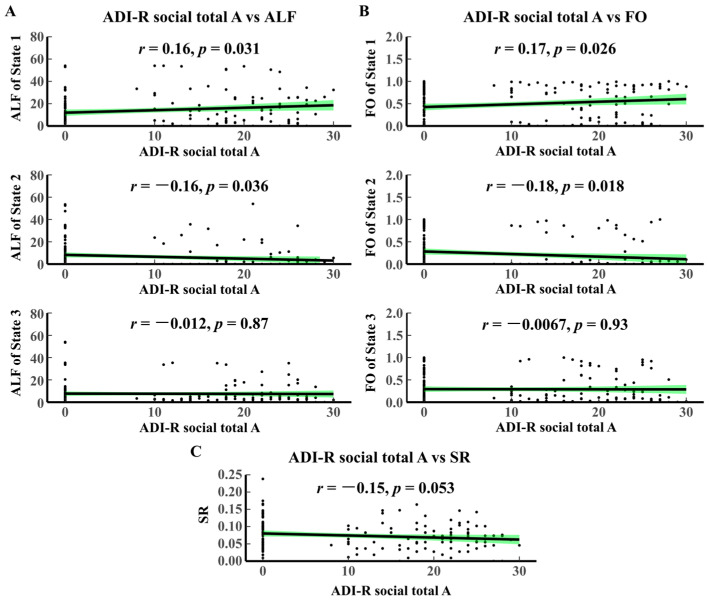
Correlation analysis between ADI-R and HMM dynamic temporal attributes. (**A**) ADI-R social total A vs. ALF. (**B**) ADI-R social total A vs. FO. (**C**) ADI-R social total A vs. SR. The black dots represent the value of HMM time-varying characteristic indicators corresponding to different clinical scale scoring values. The green area in each figure represents confidence intervals. ADI-R, autism diagnostic interview-revised; SR, switching rate.

The correlation analyses of the temporal attributes with the ADI-R social total A scale are given in Figure 5. ALF State 1 is positively correlated with ADI-R social total A (*p* = 0.0031, r = 0.16). In contrast, ALF State 2 is negatively correlated with ADI-R social total A (*p* = 0.0036, r = −0.16). FO State 1 is positively correlated with ADI-R social total A (*p* = 0.026, r = 0.16), and FO State 2 is negatively correlated with ADI-R social total A (*p* = 0.018, r = −0.18). SR is negatively correlated with ADI-R social total A (*p* = 0.027, r = −0.17). There is no significant correlation between the FIQ scale and ALF and FO. These results further validate that State 2 is the characteristic state of ASD.

### 3.4. DG Properties

**Figure 6:**
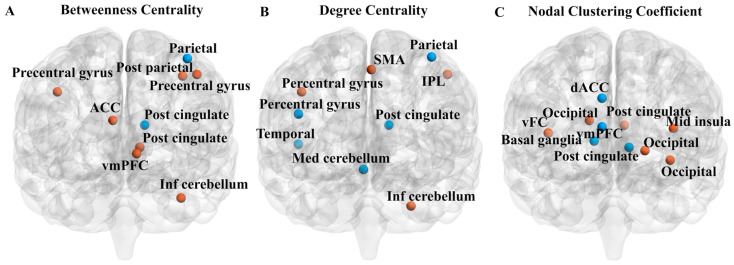
The visualization of between-group differences in (**A**) Betweenness Centrality, (**B**) Degree Centrality, and (**C**) Nodal Clustering Coefficient. Blue color indicates ASD < HC and orange color indicates ASD > HC. ACC, anterior cingulate cortex; dACC, dorsal anterior cingulate cortex; IPL, inferior parietal lobe; SMA, supplementary motor area; vFC, ventromedial frontal cortex; vmPFC, ventromedial prefrontal cortex.

The spatial-temporal variability analysis of the global and nodal attributes was conducted using DG theory. The global attributes do not have significant between-group differences. The statistical analysis results for three node attributes are shown in Figure 6 (see Table 2 for detailed node information). BC varies significantly in the VAN and default mode network (DMN). DC varies significantly in DMN and VN. The nodal clustering coefficient significantly changes in DMN and VN. The SCNs of several ROIs also differ considerably in BC and DC. These results verify the changes in the FC and network topological properties in ASD analyzed by HMM.

## 4. Discussion

Based on the ABIDE dataset, we explored the dynamic complexity of the spatial-temporal patterns of brain activity in ASD by using a data-driven HMM analysis approach on whole-brain networks and a topological DG theory analysis. Also, we analyzed the time-varying specificity between ASD and HC. The HMM analyses reveal three brain states in ASD and demonstrate the reconfigurability of HMM states in the temporal dimension. The correlation between time-varying properties and ASD behavior scores proves that State 2 is the characteristic state of ASD. The DG theory analyses validate the HMM results. The VN, VAN, and DMN of ASD produce significant changes, indicating that these network topological properties may have substantial spatial-temporal variability in resting-state brain networks [58,59,60,61].

Three global temporal characterizations of HMM states were obtained in this study: ALF, FO, and SR. These features are the most common and effective time-dynamic indicators. Significant differences were found between ASD and HC in the state tissue patterns. Consistent with previous findings, in ASD patients, ALF is significantly reduced in non-characteristic State 1, and FO is enhanced considerably in characteristic State 2 [62,63,64]. We found that ASD has a significantly higher probability of occurrence in State 1 with the weakest connectivity and is less likely to occur in State 2 with the most robust connectivity compared to the HC group. Recent studies have found that sustained connectivity in ASD patients may limit their ability to transition quickly from one brain state to another and is negatively correlated with processing speed [65]. Meanwhile, cognitive impairment in ASD may be related to changes in brain state [66]. Additionally, the correlation results between ADI-R and ADOS are consistent with the above results, indicating that State 1 does not correlate with ASD, and State 2 has a significant correlation with ASD, i.e., the lower ALF or FO of State 2, the easier it is for the brain to enter the characteristic pattern of ASD. State 2 is significantly characterized by VAN, VN, and SCN enhancement. Previous studies reported that ASD establishes more robust functional connectivity between VAN and multiple brain regions [67,68], which leads to more severe pathology, and that the degree of hyper-functional connectivity between VN and other networks is associated with more significant autism symptoms [69].

Furthermore, we used the DG theory method to assess the dynamic topological characteristics of the dynamic brain networks, and the network patterns with significant differences in node attributes have apparent coincidence with the HMM results. Three node indicators were obtained: BC, DC, and the node clustering coefficient. A brain regional node with high BC is potentially an information bottleneck [70]. The significantly increased BC in the DMN of ASD indicates a hyperconnected internal brain network. DC indicates the state and importance of the node in the brain network [71]. The significant decrease in DC of VAN and VN in ASD patients indicates a weakened centrality of nodes in these networks. The node clustering coefficient measures the degree of tightness between nodes in the network, and the increased clustering coefficient in the VN of ASD patients means that the nodes in VN have higher fault tolerance.

Although there are no significant between-group differences in our global metrics of small-world properties and global efficiency, significant changes in node attributes are observed in DMN, VAN, and VN. Specifically, there are significant differences between ASD and HC in the anterior cingulate cortex (ACC), ventral prefrontal cortex (vmPFC), and insula. There is a significant overlap between brain regions related to social cognition and default pattern networks. The DMN has been associated with rumination, self-thinking processing, and emotion assessment [72]. The inherent hyper-connectivity within DMN in ASD or the strong connections between DMN and other networks may lead to “network isolation” [73,74], limiting complex social behavior. Poor integration of DMN function may be the reason for the decline in self-referential processing ability in patients with autism. DMN also promotes preparedness for environmental changes, and abnormalities in DMN may be a reason for poor environmental awareness and response in ASD patients [75]. Due to VAN being a critical factor in attention transfer mechanisms, this network is highly correlated with many other brain functional regions. The significant changes in VAN lead to anxiety symptoms in ASD. The VN connects other cortical sensory, attention, and cognitive processing networks [76]. The abnormality of VN indicates that ASD has defects in processing external information. The altered regional topology of VN in ASD may be associated with social cognitive deficits in non-verbal communication in ASD, such as judging motor intentions and processing emotional faces [77].

Previous studies have reported a decrease in FC between DMN and other regions in ASD patients [78] and found that this abnormality can be identified as a neural functional marker of social disorders in ASD [79]. Our research findings emphasize the potential role of the networks mentioned above in the pathophysiological mechanisms of ASD, supplementing our understanding of ASD brain function from a dynamic perspective. Rashid et al. found that longer residence time is associated with global disconnection in adolescents with higher autism characteristics [80]. Our study find that ASD patients have shorter residence time and lower frequency in weakly connected states, and their brain activity is more likely to enter a strong connectivity state, which limits their ability to engage in complex social behaviors, providing additional evidence for evaluating the impact of psychiatric ASD. HMM analysis is a probabilistic representation of the state and transition space of the entire brain network, which may provide new insights into the neural mechanisms of ASD.

This study has some limitations. Firstly, we did not explore the impact of gender differences on our findings. Based on our criteria of selecting participants from the ABIDE I multi-site dataset, there was a significant gender imbalance. There were more males than females in both ASD and HC groups. As is well known, ASD is more common in males than females, and gender differences do have a significant impact on fMRI-based brain functional connectivity research and clinical manifestations [81]. However, it has been reported that based on the ABIDE I multi-site dataset, gender has not been observed to have a significant impact on hyperconnectivity or low connectivity in brain [82]. As the current evaluation measures for male and female ASD are designed and standardized using male samples, the effectiveness and sensitivity of the impact of gender differences are still questionable [83]. Secondly, the interference of noise on brain imaging signals caused by scanning equipment and subjects cannot be eliminated completely even with strict acquisition control and relatively complete data preprocessing steps. The heterogeneity of multi-site data may also affect the relevant results.

## 5. Conclusions

In conclusion, we used HMM and DG theory to investigate the DFC changes and topological features between ASD and HC. The HMM analyses reveal significant differences in VAN, VN, and SCN between ASD and HC, and the DG analyses verify these findings. HMM provides rich information about brain dynamics in the time dimension and compensates for the lack of spatial-temporal characteristics of brain activity in graph theory. Our study shows that ASD has lower residence time and occurrence frequency in the weak connectivity state. ASD is more likely to enter the strongly connected state with high occurrence frequency, leading to the isolation of brain networks and changes in topological features of DMN, VAN, and VN, aggravating the disease of ASD. These results were confirmed by correlation analysis between HMM time-varying properties and ASD behavior scores. By identifying abnormal changes in FC and topological features, potential neuroimaging markers are provided for the diagnosis of ASD. The complex structure of brain networks is the basis of cognitive function and behavior, and abnormalities in brain networks can lead to neurological diseases. Based on functional magnetic resonance imaging (fMRI), this study used two data-driven approaches to study brain networks affected by autism disorders, providing new insights for identifying neuro-biomarkers and developing interventions for brain nodes to some extent.

For future work, the impact of gender on the spatial-temporal characteristics of the ASD brain is worth exploring in depth. The application of the methods proposed herein to task-state fMRI data of ASD and HC or the addition of drug therapy is also worth studying. The results and conclusions of this study are provided based on our specific implementation framework, including datasets, charts, statistical analysis, and classification methods. The generalizability of these findings can be further explored in more datasets (such as ABIDE II). Global graph theory metrics can also be analyzed to describe the characteristics of the entire brain. Using machine learning methods to further explore the value of this study may bring new scientific significance.

## Figures and Tables

**Table 1:** Demographic and clinical characteristics of the subjects included in this study.

	ASD (n = 87)	HC (n = 90)	Group Comparison (*p*-Value)
Sex (M/F)	75/12	70/20	0.1468
Age	11.78 ± 2.74	11.96 ± 2.64	0.6455
Handedness (L/R/M)	12/72/3	6/80/4	-
FIQ	103.0 ± 17.07	108.88 ± 12.02	0.0086 (*t* = −2.656)
ADI-R			
Social score	19.74 ± 5.56	-	-
Communication score	15.54 ± 5.01	-	-
Gotham RRB score	6.59 ± 2.40	-	-
ADOS			
Total score	11.75 ± 3.37	-	-
Social score	8.23 ± 2.29	-	-
Communication score	3.52 ± 1.55	-	-
Gotham RRB score	2.77 ± 1.90	-	-

Note: n is the number of subjects, and *t* is the statistical value of a two-tailed, two-sample *t*-test. Data are mean ± SD, M/F, male/female, L/R/M, left/right/mix. ADI-R, Autism Diagnostic Interview-Revised; ADOS, Autism Diagnostic Observation Schedule; ASD, autism spectrum disorder; FIQ, full intelligence quotient; HC, healthy control; RRB, restricted and repetitive behaviors.

**Table 2:** Statistical analysis results of node attributes.

	Label	*p*-Value	*t*-Value	ROI	Yeo 7
Betweenness Centrality					
	5	0.0013	3.2659	vmPFC	DMN
	8	0.0309	2.1758	ACC	VAN
	17	0.0189	2.3691	Post cingulate	DMN
	26	0.0445	−2.0238	Post cingulate	VAN
	50	0.0297	2.1917	Post parietal	DAN
	97	0.0224	2.3041	Precentral gyrus	VAN
	101	0.0028	3.0292	Precentral gyrus	DMN
	106	0.0370	−2.1021	Parietal	FPN
	153	0.0251	2.2588	Inf cerebellum	SCN
Degree Centrality					
	26	0.0094	−2.6245	Post cingulate	VAN
	51	0.0125	2.5250	IPL	SMN
	93	0.0400	2.0687	SMA	DMN
	99	0.0086	−2.6576	Precentral gyrus	DMN
	101	0.0289	2.2035	Precentral gyrus	DMN
	115	0.0288	−2.2043	Parietal	VN
	124	0.0148	−2.4622	Temporal	VN
	148	0.0283	2.2108	Inf cerebellum	SCN
	156	0.0218	−2.3143	Med cerebellum	SCN
Nodal Clustering Coefficient					
	4	0.0215	−2.3201	vmPFC	DMN
	17	0.0248	−2.2644	Post cingulate	DMN
	23	0.0123	2.5290	Post cingulate	FPN
	61	0.0474	−1.9970	dACC	SMN
	67	0.0265	−2.2378	Basal ganglia	SMN
	92	0.0245	2.2692	vFC	VN
	104	0.0333	2.1448	Mid insula	DMN
	121	0.0272	2.2270	Occipital	DMN
	122	0.0407	2.0623	Occipital	VN
	127	0.0178	2.3925	Occipital	VN

Note: ACC, anterior cingulate cortex; dACC, dorsal anterior cingulate cortex; DMN, default mode network; IPL, inferior parietal lobe; SCN, subcortical network; SMA, supplementary motor area; SMN, sensory and motor network; VAN, ventral attention network; vFC, ventromedial frontal cortex; vmPFC, ventral prefrontal cortex; VN, visual network.

## Data Availability

The original data presented in the study are openly available in ABIDE I at: http://fcon_1000.projects.nitrc.org/indi/abide/, accessed on 10 June 2023.
